# In-Situ Measurements in Microscale Gas Flows—Conventional Sensors or Something Else?

**DOI:** 10.3390/mi10050292

**Published:** 2019-04-29

**Authors:** Juergen J. Brandner

**Affiliations:** Staff Position Microstructures and Process Sensors (MPS), Institute of Microstructure Technology (IMT), Karlsruhe Institute of Technology (KIT), Hermann-von-Helmholtz-Platz 1, 76344 Eggenstein-Leopoldshafen, Germany; juergen.brandner@kit.edu; Tel.: +49-721-6082-3963

**Keywords:** miniaturization, gas flows in micro scale, measurement and control, integrated micro sensors, advanced measurement technologies

## Abstract

Within the last few decades miniaturization has a driving force in almost all areas of technology, leading to a tremendous intensification of systems and processes. Information technology provides now data density several orders of magnitude higher than a few years ago, and the smartphone technology includes, as well the simple ability to communicate with others, features like internet, video and music streaming, but also implementation of the global positioning system, environment sensors or measurement systems for individual health. So-called wearables are everywhere, from the physio-parameter sensing wrist smart watch up to the measurement of heart rates by underwear. This trend holds also for gas flow applications, where complex flow arrangements and measurement systems formerly designed for a macro scale have been transferred into miniaturized versions. Thus, those systems took advantage of the increased surface to volume ratio as well as of the improved heat and mass transfer behavior of miniaturized equipment. In accordance, disadvantages like gas flow mal-distribution on parallelized mini- or micro tubes or channels as well as increased pressure losses due to the minimized hydraulic diameters and an increased roughness-to-dimension ratio have to be taken into account. Furthermore, major problems are arising for measurement and control to be implemented for *in-situ* and/or *in-operando* measurements. Currently, correlated measurements are widely discussed to obtain a more comprehensive view to a process by using a broad variety of measurement techniques complementing each other. Techniques for correlated measurements may include commonly used techniques like thermocouples or pressure sensors as well as more complex systems like gas chromatography, mass spectrometry, infrared or ultraviolet spectroscopy and many others. Some of these techniques can be miniaturized, some of them cannot yet. Those should, nevertheless, be able to conduct measurements at the same location and the same time, preferably *in-situ* and *in-operando*. Therefore, combinations of measurement instruments might be necessary, which will provide complementary techniques for accessing local process information. A recently more intensively discussed additional possibility is the application of nuclear magnetic resonance (NMR) systems, which might be useful in combination with other, more conventional measurement techniques. NMR is currently undergoing a tremendous change from large-scale to benchtop measurement systems, and it will most likely be further miniaturized. NMR allows a multitude of different measurements, which are normally covered by several instruments. Additionally, NMR can be combined very well with other measurement equipment to perform correlative *in-situ* and *in-operando* measurements. Such combinations of several instruments would allow us to retrieve an “information cloud” of a process. This paper will present a view of some common measurement techniques and the difficulties of applying them on one hand in a miniaturized scale, and on the other hand in a correlative mode. Basic suggestions to achieve the above-mentioned objective by a combination of different methods including NMR will be given.

## 1. Introduction

The interest to gas flows in miniaturized systems has grown tremendously in the last couple of years. Driven by areas such as the automotive industry, semiconductors, the chemical and pharmaceutical industry, modeling, precise measurement and control of the flow of gaseous compounds, mixtures and reactive systems through mini- and micro-structured devices gained importance for various applications like heat transfer [1,2,3,4,5,6,7,8,9,10,11,12,13,14,15,16], gas-liquid or gas-solid contacting [17,18,19,20,21,22,23,24] and chemical reactions [25,26,27,28,29,30]. Amongst the correct design and manufacturing of the miniaturized devices, all the named topics need precise measurement and control of the processes taking place inside microstructures. The final objective of these efforts is to provide an almost comprehensive description of the process to be able to model and simulate as well as to predict by software. The following description will focus on measurement and close out control, to make the process is not too exhaustive.

**Figure 1:**
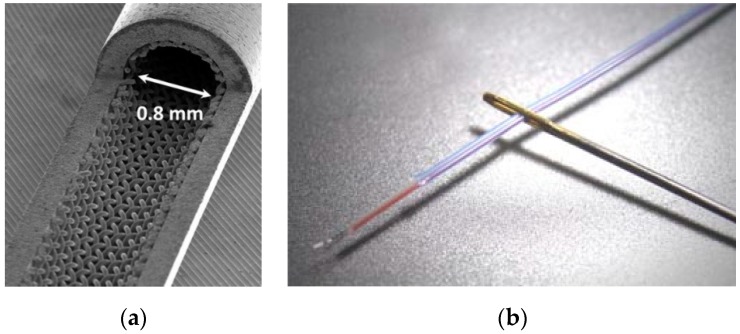
Microstructure fluidic device. (**a**) Micro heat pipe, opened to see the inside structure. Inner diameter is 0.8 mm. From: KIT-IMVT; (**b**) Miniaturized Type K thermocouple. Although this sensor has an outer diameter of 0.4 mm only, a major part of the structure in (**a**) would have been blocked. Picture from: www.ninomiya-ew.co.jp.

While measurement of gaseous flows in macroscale is not trivial but manageable, it turns out to be much more problematic in the micro scale. There are several reasons for this. The fact that gas is a compressible medium per se makes it more difficult to measure certain behavior and parameters of a flow inside confined or miniaturized systems [31,32,33,34,35]. Additionally, the scale down of conventionally-sized macro scale tubes or channels into the micro scale makes it more complex to measure. This is, on one hand, due to sensors which are simply too large to be inserted into the micro devices. Figure 1a,b show examples for this. For temperature measurement in macro-scale tubes, a thermocouple is simply located into the flow, measuring the gas flow temperature. This is changed by scaling down the tube diameter. While Figure 1a shows a mini heat pipe cut open, Figure 1b provides a view to a so-called “micro-thermocouple”. It is quite obvious that the thermocouple will block the inner diameter to a major extent and, therefore, completely change the fluidic behavior. Thus, no precise measurement would be possible. More problems occur due to the low density of gases, the change in viscosity, the small specific heat capacity and the need for increased leak-tightness of microstructures while handling gases (and here, depending to the gas, the acceptable leak rate Q_L_ can vary in orders of magnitude!). However, in many cases Micro Electromechanical Systems (MEMS) have been applied as measurement tools to be implemented into microstructure devices [33]. While this is often a practical solution, in other cases it is not, as will be shown in the later discussion.

Nevertheless, from 1964 until today, roughly about 45,000 papers have been published on miniaturized gas sensors and sensor systems [36]. A large variety of relevant technologies is available which will not be described in this paper in detail, more can be found in comprehensive textbooks or in reference [37].

The following will present an overview on parameters of gas flows in microstructures to be measured as well as measurement methods for these. The presented overview is by no means complete. The envisaged field of measurement is very much in flux; thus, new technologies and improved methods are rapidly developing, especially by improving the sensitivity and selectivity of the measurements. One of these developments is the use of nanomaterials for sensing opportunities. Here, numerous different technologies based on nanostructured materials or nanostructures in specific materials have been described and are used now for sensing in gases. Examples are given in references [38,39,40,41,42,43,44,45,46,47]. The sensitivity and selectivity of nanostructure/nanomaterials sensors has improved significantly in the last few years [48,49,50].

Another trend is a combination of sensing elements in micro or nano scale for measurement of biological parameters or environment (biosensing, [51,52,53,54,55,56]). This field is relatively new, and lots of developments are to expect in the next future.

As mentioned before, an additional point is the correlation of a multitude of measurement methods to achieve a more dense “information cloud” and, therefore, reach a better understanding of the effects and behavior in gas flows. This leads to the necessity of *in-situ* and *in-operando* combination of several measurement methods with similar (ideally: identical) timely and spatial resolution. All measurements have to be taken simultaneously and at the same location to provide the highest possible information density of the process taking place at this point. As an example, for such a process a heterogeneously catalyzed gas phase reaction could be taken. Here, gas flow, gas composition, catalyst-gas-surface interaction, temperature, pressure and product concentration should be measured at the same time and the same location, to name just a few of the parameters of such a process.

The data obtained from each of such monitoring systems will then be correlated to those of all others, in time as well as in space, to get a description of an n-dimensional parameter space that is as comprehensive as possible. Within the overview presented here, several possible technologies to correlate measurements are very briefly and rudimentarily described. Possibilities, advantages and disadvantages of each method are presented, and a possible use in combination with other methods is evaluated. This overview is not intended to give a vast description of the measurement methods, their advantages and disadvantages, their working principles or underlying functionalities—this is left to much more specified papers or textbooks. This collection, without delving deeply into details of the methods themselves, should provide a quick overview for possible correlative measurement technologies only. For this objective, the following section deals with the parameters which most regularly are measured, as well as with some most common methods for those and with scaling, the problem of miniaturization itself [57,58,59].

## 2. Parameters, Measurement Methods and Scaling

Numerous parameters of gas flows in micro scale can be measured. However, many of them are only useful in specific applications. Amongst those are parameters like chemical reactivity, pH, solubility in liquids, speed of sound, etc. This publication will focus on more common parameters like temperature (or temperature difference), heat flux, pressure (or pressure difference), mass flow, flow velocity, mixture composition and concentration (concentration gradients, respectively) of species inside micro scale gas flows.

The measurement methods can in general be split in electrical measurements using conventional wire systems [60,61], electrical measurements using micro-electromechanical systems (MEMS) [62], spectroscopic measurements or optical measurement methods [63]. There are numerous papers on each of those different measurement methods, thus, it shall not be the objective of this publication to provide more detailed information on them.

Wire-system based methods use conventional sensors which are, in many cases, macroscopic. In general, these are thermocouples or thermistors (see below), pressure sensors, mass flow meters and similar, which are well known measurement devices in style as well as in behavior. These sensors are mounted on the process tubing, e.g., thermocouples before and after a possible reaction vessel as well as pressure sensors or mass flow meters. An *in-situ* or *in-operando* monitoring is hardly possible.

MEMS-based measurements show the possibility to be implemented for *in-situ* and *in-operando* measurements. Pressure sensors, temperature sensors, flow sensors and others more are available off the shelf. They are, in general, Si-based, small, provide a short response time, good reliability and long lifetime. Additionally, most of those systems are cheap to produce. However, they need to be coupled with a visualization module or a data logger to make the measurements available for the operator. This is regularly done by wiring. There are some cases of wireless MEMS sensors [64]. However, due to the continuous developments in semiconductor technology, wireless solutions are becoming more and more popular, and their use is greatly enhanced by, i.e., radio frequency identification (RFID) and near field communication (NFC) technology [65,66,67,68,69].

What was mentioned above for wire-based electrical sensor in principle holds also for optical sensors, where either an optical fiber is implemented into the measurement location, or an active optical component (light emitting diode LED, laser or other light source) is integrated there—in many cases an optical detector like a photo diode or similar could also be used. Examples are presented in references [70,71,72,73,74,75,76].

Most of the measurement methods of the sensors named above have been derived from macroscopic standards, adapted and improved for the mini scale and then applied and even further revised for the micro scale; and almost all of them (but the wireless MEMS devices) share the same problem of scaling. This holds for all type of sensors, whether electrical or optical.

**Figure 2:**
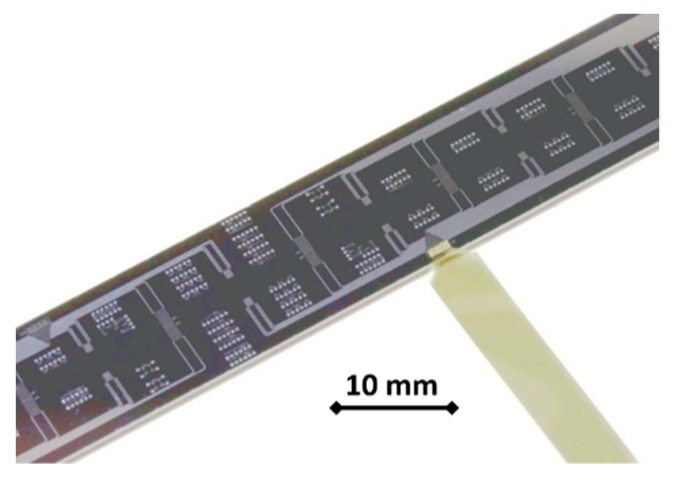
Silicon microstructure chip for temperature measurement in a gas flow. The chip is inserted into a sealed housing and forms the fourth side of a rectangular microchannel. Numerous thermopiles have been implemented into the chip, to measure the temperature of a gas flow in combination with precision resistors [78]. www.gasmems.eu.

Scaling a measurement method simply down from macro to micro scale will normally not generate the desired results. Either is the fluid influenced too strongly by the measurement method (as was shown in the example given in Figure 1), or the precision of measurement suffers from the miniaturization. It is, in many cases, not easy to decide where in the fluid flow to place the sensors to obtain correct measurements. A good example is the measurement of temperatures. If a MEMS-based measurement system is placed into the sidewall of a microfluidic system, the wall temperature is measured, or maybe the temperature of the gas flow near the wall. The same result will be obtained with an optical element placed inside the sidewall of a microfluidic system. The temperature in the core of the flow is not determined in any way. Moreover, the MEMS system may alter the wall-gas interaction (because the material might be different), and therefore not even provide a representative measurement signal for the sidewall temperature of the gas flow [77]. This might not be so much the case for the optical sensor. Figure 2 shows an example of a silicon MEMS sensor system used for temperature measurement inside a microchannel gas flow under ambient or slightly rarefied conditions [78].

If the process is well known, the core temperature of the flow might be calculated correctly. Not knowing the process precisely, it might be necessary to measure the core flow temperature—which is possible with a sensor implemented into the flow. Placing a very thin wired sensor or a thin optical fiber inside the flow core may lead to a more precise measurement of the temperature there, but also could lead to a deflection of the measurement sensor due to insufficient stiffness, or to generate local turbulences and eddies and, therefore, disturb the flow. The same holds for a wireless sensor, which has to be mounted and fixed somehow in the flow. In any case, scaling down fluidic systems for gas flows into the micro scale needs a very careful consideration of the location and the precision of the sensors applied. This holds for all parameters to be measured.

### 2.1. Temperature Measurement

One of the most common tasks in gas flows is the measurement of temperatures, as mentioned above. At the same time, it is a relatively complex measurement, which is, in many cases, underestimated [60,79,80,81,82,83,84]. In the following, some possible methods will be described and evaluated as a general example for other parameters to be measured at a micro scale.

#### 2.1.1. Measurements Using Conventional Intrusive Sensors

Conventional sensors in this case means either thermistors or thermocouples. Optical sensors shall not be considered here, because they are more delicate to handle in this case and are generally more expensive [85,86,87]. Both thermocouples and thermistors are commercially available in various shapes, sizes and forms, which makes them flexible and handy tools. Moreover, efforts to obtain some results are limited. In both cases, wired standard solutions as well as wired MEMS are available.

Thermistors are temperature-dependent electrical resistors. They can be separated in devices with a positive temperature coefficient retrieving a higher resistance with increasing temperature, or a with negative temperature coefficient, which lowers the resistance by increasing temperature. In any case, the dependency between the change of resistance and the temperature is very linear (or can be linearized in an easy way) [88]. The most common example is the PT100, a platinum resistor with a nominal value of 100 Ω at standard conditions (variations with 10 Ω or 1000 Ω are also common). The PT-sensor is used as part of a Wheatstone bridge circuit, which makes a very precise measurement possible [89].

Temperature sensors based on the resistor principle are wide-spread in any application, because they can be manufactured as discrete devices as well as integrated circuits in semiconductor technology. Thus, extremely small sensors can be generated using standard semiconductor manufacturing processes. This also holds, in some cases, for the second common temperature measurement principle, which is thermocouples.

A thermocouple is an electrical device consisting of two dissimilar electrical conductors forming electrical junctions at differing temperatures. A thermocouple produces a temperature-dependent voltage as a result of the thermoelectric effect, and this voltage can be interpreted to measure a temperature [78,90]. Some types of thermocouples can also be produced in a micro- or even nano scale by use of semiconductor technology, while others cannot [37]. However, the two main disadvantages of thermocouples are their limited signal strength and the need for a reference temperature. While the signal strength can be enhanced by creating thermopiles (numerous thermocouples circuited in a row, see Figure 2), the reference temperature need cannot be avoided. This fact makes it difficult to apply thermocouples in miniaturized equipment, which regularly shows a more or less isothermal behavior, short distances in the mm or µm range and very short temperature spreading times. Thus, the difference between reference temperature and measured temperature might be diminished rapidly.

Both measurement principles mentioned above are regularly carried out with wired sensors. Wireless temperature sensors, especially in micro scale, are not so widely common yet, but will be more common in the future [91,92,93]. However, all the sensors presented in this subsection need a wire connection to the outer environment, thus, sealings and interconnections are necessary. This might be, depending on the supervised process, a source of leakage and uncertainty. Therefore, wireless measurement would be a better option, as was mentioned before. Additionally, it was mentioned that introducing a sensor into the gas flow would disturb the flow mode or even change the process parameters significantly. Thus, non-intrusive methods have to be used.

#### 2.1.2. Measurements Using Non-Intrusive Methods

As the example of the use of conventionally sized resistors or thermocouples (presented in Figure 1b) shows, this is, in many cases, not an option, mainly for size reasons as described before. Thus, it has to be asked which other techniques and methods could be used for such a simple measurement task such as retrieving the temperature?

One option is the use of non-intrusive optical systems (i.e., infrared (IR) radiation, ultra violet (UV) fluorescence, others) or ultrasound [94,95,96]. Here, surface temperatures are measured, providing a non-intrusive possibility to acquire data on the thermal state of a body. However, these methods have several disadvantages. They are not applicable for all cases, since certain materials are not transparent for IR radiation (i.e., common glasses, metals etc.) or reflect or damp-out ultrasound very strongly (i.e., dense liquids, metals etc.). In such cases, a wrong signal, a strongly reduced signal or no signal at all can be obtained, leading to a very low signal-to-noise ratio (SNR). When this occurs, a measurement might be useless. A further disadvantage is that the measured signal represents the surface temperature of a device obtained by conduction, convection or radiation from the inner side, but not the core temperature. This means the inner parts of a body could differ significantly in temperature from the outer surface. This outcome cannot be seen and measured by the chosen measurement technology. The same holds for other measurement technologies that use optical fluorescence to measure temperatures [97].

Another option is Raman spectrometry [98]. With this technique, temperature distributions inside of miniaturized structures are easy to measure if the surrounding area is transparent for the Raman light. All the limits named before for IR and ultrasound measurements hold here also, because this is still an optical method. Efforts are high, and the Raman systems are expensive and complex to handle. If all these drawbacks are given for measurement of an all-day parameter like temperature, does this also hold for other measurement needs like pressure, flow, density or more complex sets of parameters like local concentration? Furthrmore, what might be feasible in terms of *in-situ*, *in-operando* and correlated measurements?

### 2.2. Measurements of Other Parameters

**Figure 3:**
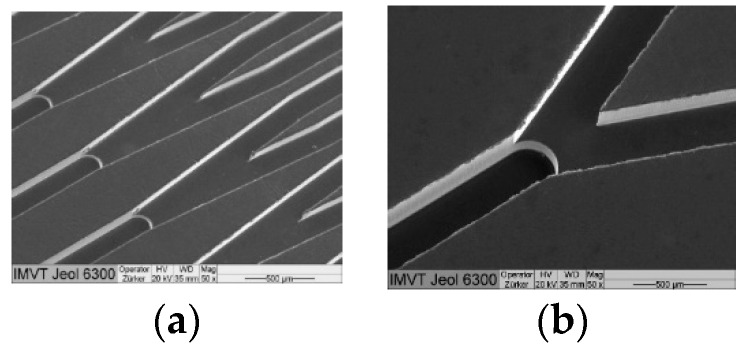
Bifurcative microchannel distribution system for liquids and gases. (**a**) Overview to a major part of the system; (**b**) Magnification of one of the splits from (**a**). A step between the single incoming channel and the two outgoing channels is clearly visible, which is due to manufacturing. Reproduced with permission from [111], published by J-STAGE, 2012.

As pointed out in the previous sections, measurements of several parameters lead to application of different techniques at a single process. The process pressure is measured with pressure transducers, in most cases located outside the process vessel. The process flow through tubes or vessels is obtained by anemometers or similar systems. Electrochemical sensors are used to measure pH or conductivity. Viscosity is obtained with a rheometer (in general, outside of the process run), and so on. Additional analytical methods like gas chromatography (GC) [99,100], infrared (IR) spectrometry [101], ultraviolet (UV) spectrometry [102], the above mentioned Raman spectrometry [103], mass spectrometry (MS) [104,105] or Nuclear Magnetic Resonance spectroscopy (NMR) [106,107,108,109,110] are used to determine the components of the mixture, depending on the applicability of the respective method to the gas. Visualization of flow processes can be obtained by, for example, high speed videography [111,112,113]. An example of a microstructure used for high speed videography is given in Figure 3. 

All of these systems work, in most cases, independently, are time consuming and costly. *In-situ* measurements or *in-operando* measurements inside of micro scale systems are possible, but rarely done, in most cases the analysis is performed offline with a specific sample taken from the process flow. A correlation of results of these analytical methods is used to gain more complete process information, as was proposed in the introduction section, with measurement results of the process parameters obtained as described before being at least difficult [114,115,116], in many cases it is almost impossible.

Thus, it might be a good idea to have a combination of several complementary measurement and analytical systems which can provide all the information wanted within a single measurement campaign at once.

### 2.3. Combined and Correlated Measurement System

**Figure 4:**
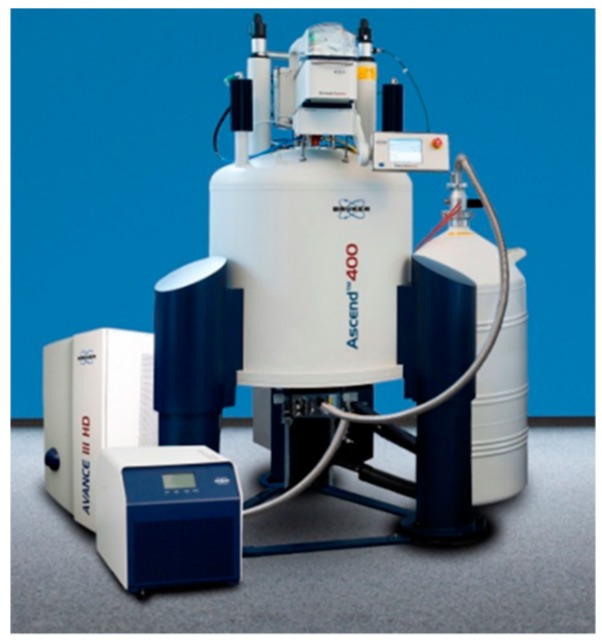
400 MHz (9.4 T) NMR system of the Bruker company.

The objective mentioned above, namely to measure *in-situ*, *in-operando*, simultaneously at the same location, can be obtained by combining several measurement and analytical systems. However, aside of the tremendous efforts to be undertaken, this will result most likely in a spatial correlation only. Due to different measurement time constants of the different sensors and systems, a timely correlation is very unlikely to be obtained. All the methods named above show drawbacks for *in-situ*, *in-operando*, combined and correlated measurements. While Raman spectroscopy will need an optical access, IR spectroscopy as well as UV spectroscopy will need additionally specimen active in the named wavelength region. *In-situ* and *in-operando* GC or GC-MS is possible (see i.e., reference [117]), but in general is done in a macro scale. To scale those techniques down into micro scale might be feasible but is generally not available as an option yet. NMR measurement systems [110] are, in general, big machines with super-cooled magnets and a huge bunch of external equipment, which is one of the major drawbacks of this technology. Figure 4 shows an example of a 400 MHz (9.4 T) NMR device made by the Bruker company (Billerica, MA, USA).

However, with an NMR spectrometer like this, a sample can be characterized for various parameters in a single measurement campaign, correlating time and location with each of the separate measurements taken. The question arising now is whether such a system is able to characterize the desired parameters with a spatial and time resolution that is high enough to be useful in a micro scale.

For the spatial resolution, a value of about 10 µm is given in literature (see i.e., reference [118]). The time resolution is named to be around 20 ms (see i.e., reference [119]). This result is assumed to be valid for a multitude of measurement objectives performed by NMR, spatial as well as time resolution. The literature suggests that this result is in principle good enough for running NMR systems as well-chosen measurement and analytical tools for numerous applications, ranging from chemistry to materials research, pharmacy and food engineering to biomedical applications or physics. However, it has to be considered that many measurements will not be done after the 20 ms given above, but instead take much longer and average a multitude of single measurement shots.

Thus, beside the drawbacks of size, costs and running efforts for such a device in terms of liquid helium and nitrogen for cooling down the magnets etc., it is not clear yet whether all desired parameters can be measured in reasonable time, or if even all parameters important for a process to be characterized can be measured. Aside of this, not all materials can be used with NMR. The technology is very selective, but is limited to non-magnetic materials for devices. If process parameters can only be reached using a vessel made of magnetic material, then NMR measurement methods fail due to the magnetic properties of the device. Another drawback is that not all desired parameters can be measured directly, some can only be retrieved indirectly.

While the measurement of temperature with NMR is in some cases precise and simple [120], the process pressure can only be retrieved by pressure-sensitive materials [121]. Viscosity, density, mass flow and phase, phase changes or particle content can be measured directly (i.e., reference [122]), as well as the electrochemical potential of compounds (see reference [123]), concentrations of single mixture compounds [124] or the pH of a mixture. 

**Figure 5:**
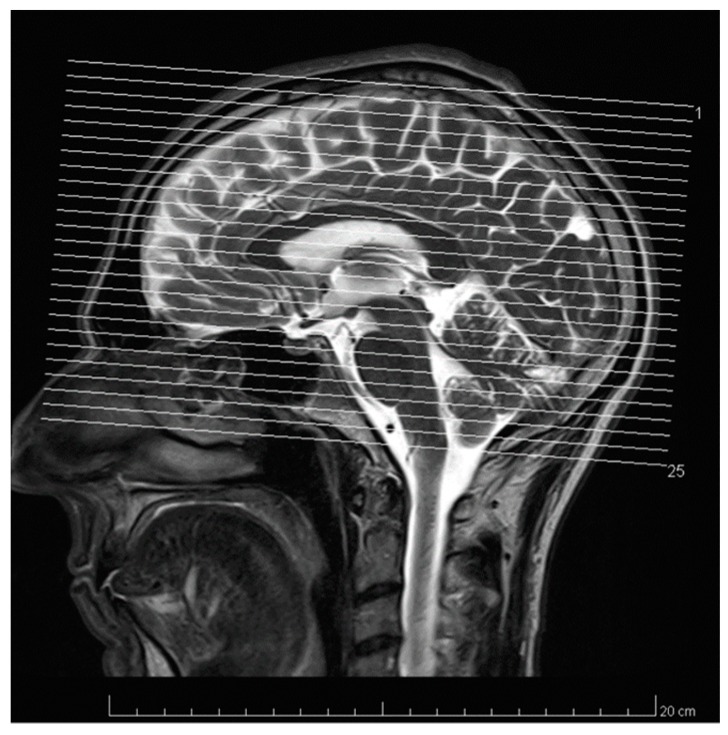
Magnetic resonance tomography (MRT) cut picture of a human head as an example for the MRI possibilities. From: Private.

Additionally, this technology allows us not only to measure different parameters non-intrusively in very small confinements like micro channels, but also in living tissues [125,126]. This is an add-on not provided by a multitude of the other techniques presented before. Another add-on is the possibility to visualize processes by magnetic resonance imaging (MRI, see reference [127]). An example of this is presented in Figure 5, showing a cut through a human head and imaging the brain.

Thus, NMR seems to provide the possibility to cover lots of measurement tasks of other methods in a single machine, being non-invasive, non-intrusive and non-detrimental to the measured object. With all the possibilities provided here, a discussion is needed on at least two topics: is measurement with an NMR correlative? Moreover, is NMR measurement the best possible solution for every measurement task?

## 3. Discussion

**Figure 6:**
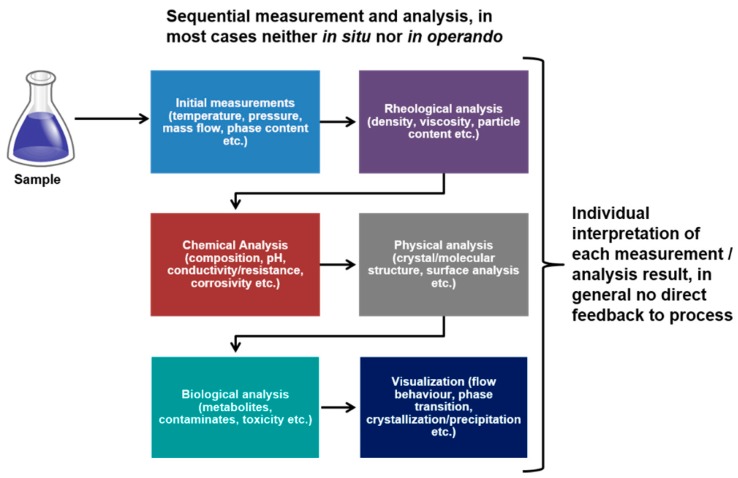
Current state-of-the-art sequential measurement and analysis method. The timely and locally separated measurement makes it difficult or even impossible to correlate measurement results to each other and retrieve by this deeper insight into the underlying mechanisms.

As was pointed out in Section 2, lots of different methods for measurement of process parameters are available and have been explored. Miniaturization of process systems results in major problems for scaling them down, as was mentioned and described before. However, with larger efforts, smart design and good planning of experiments, the scaling down of measurement systems and obtaining precise results of the process parameters is possible to a certain extent, using various methods for process parameter characterization. Thus, miniaturization and scaling is a problem for intrusive measurement methods, while precise measurement of the parameter distribution is a problem for non-intrusive methods. This has clearly been described in Section 2. In almost all cases, measurements are performed sequentially, having each of the measurement and analysis systems running after the one before. This type of performance, aside from the difficulties given by scaling, allows us to achieve good and reliable measurement results, but not to correlate them to each other and to obtain a more fundamental understanding of the interactions between process parameters and underlying principles. This is shown schematically in Figure 6, in which the current state-of-the-art measurement methodology is presented.

**Figure 7:**
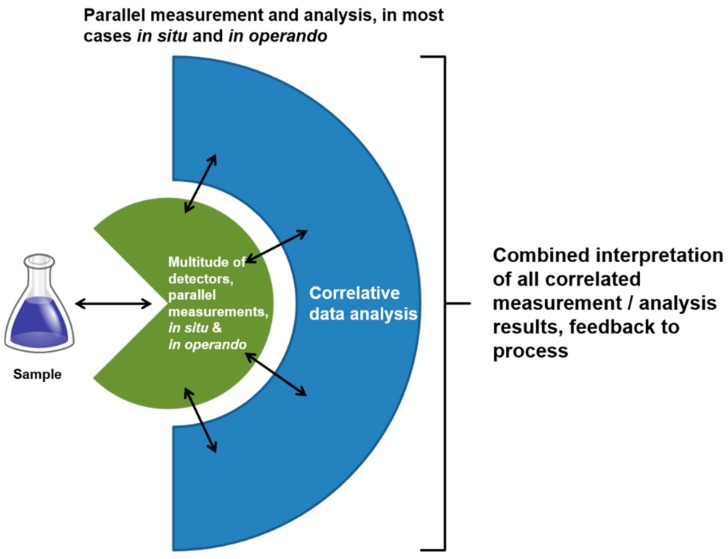
Future improved parallel measurement and analysis method. The detectors are triggered automatically in a way that they all measure at the same location, and deliver the measured data with the same spatial and time resolution. Data are then transferred to an analysis level, where they are correlated.

Thus, correlation of the measurement results is the major point to deal with. One of the future perspectives of measurement is to obtain correlated information *in situ* and *in operando* by as many different techniques as possible. Correlated measurement means timely and spatially, and with similar resolutions for all applied techniques. This is necessary to gain an exhaustive view of the examined process, to understand the underlying principles and actions, and therefore to make them accessible for modeling and simulation as well as in silico prediction and optimization. The latter will lead to major reductions in resource consumption. Figure 7 shows a scheme of a possible future measurement environment, in which a multitude of instruments are involved in parallel, obtaining means at the same time and location, measuring *in situ* and *in operando* and, therefore, delivering results which can directly be correlated. With this method, a much deeper understanding of links between actions and effects can be achieved in a more appropriate way. Thus, correlative measurement, generation of linked information and interpretation, management and feedback of connected data will be one of the main tasks in the future. The measurement structure schematically shown in Figure 7 can support this task.

Some of the measurement methods presented above are useful for stand-alone measurements only, while some can be performed simultaneously. However, the methods and techniques described in Section 2.1 and Section 2.2 can hardly be done in a correlated way. It is possible to measure temperature, pressure and chemical composition of a flow at the same position using, i.e., thermocouples, pressure sensors and Raman spectrometry. To obtain a precise time correlation by using a trigger is difficult, mainly due to different time constants of the measurement methods. Additional visualization of the process adds some more problems, because for high speed videography as described in Section 2, very high light intensities are necessary, which might cope with other optical measurement methods; therefore, a combined high speed videography—Raman measurement is not an option.

All this holds for all of the conventional methods described above. Without precise triggering, correlative measurement is most likely impossible; and even with a time trigger, the location of measurement as well as the spatial resolution most likely differs largely.

NMR makes it partly easier in this particular point. As described before, in principle a multitude of parameters can be retrieved with NMR, all of them at the same location. Thus, spatial resolution is consistent. However, it is doubtful that a timely consistence can be reached because some measurements simply take much longer than others, as pointed out before. Thus, truly correlative measurements in terms of time and space cannot be generated for the most cases, meaning this problem remains. However, the data measured by NMR are to a certain extent per se correlated, because several parameters are measured with the same method and then averaged on a time period, which is a huge advantage compared to conventional measurement equipment. Process pressure has to be measured via pressure-sensitive materials (see description above and reference [121]), which makes this a special case to be dealt with. Additionally, with MRI a handy visualization tool is available, which provides an even more in-sight view to processes and flows. Thus, many of the problems attached to conventional measurement systems apply less for NMR. However, the time correlation problem is kept, and due to the limitation of applicability to non-magnetic device materials, NMR is not a panacea. It is a good toolbox for lots of possible measurement solutions correlating the obtained data directly internally. A future vision would be to retrieve all process parameters at the same time, with the same precision, the same spatial and time resolution, *in-situ* and *in-operando*, as was requested. Thus, NMR might be a nice additional tool for certain measurement applications, but it cannot be considered to be a stand-alone tool for generation of correlated data on processes yet. There’s a need for combinations of methods, to obtain the desired “information cloud”. Only with this, a reasonable combination of different measurement and analytical tools, might a more comprehensive descriptions of processes be feasible.

Recently ideas for further correlated measurements have emerged, like MR-optical combinations, or the integration of atomic force microscopy (AFM) and NMR into a single device. Moreover, proposals on combining NMR technology with additive manufacturing, i.e., 3D printing methods, came up. Here, the characterization as well as the measurement would be integrated in a single process, allowing us to generate miniaturized systems in a way that was not even considered ten years ago.

## 4. Conclusions

Macroscale measurement of gas flow parameters with conventional technology like thermistors, pressure sensors etc. is no longer a problem, but scaling those systems down into the mini or micro scale remains a challenge. Due to either insufficient size of classical sensors or insufficient possibilities to position micro sensors in the gas flow, the measurement largely influences the process itself. Non-intrusive methods are possible, but show other limitations, like optical accessibility, the need for high power light exposure or non-magnetic equipment materials.

One of the most interesting measurement problems is to achieve timely and spatially correlated results. By using conventional methods involving thermistors, GC, MS, Raman, IR or UV spectroscopy, pressure transducers etc., this is almost impossible. Either the measurement location largely differs, or the time resolution is simply not suitable. In addition, the combination of data from different measurement sources is not trivial. The use of nuclear magnetic resonance systems (NMR), which in principle allow measurement of a multitude of parameters at least at the same location, and in most cases with the same spatial resolution, eases this problem slightly. Here, results can be obtained from the same measurement location, show a consistent data format, and can therefore be easily correlated. Even visualization is relatively easily achieved by combining regular NMR with magnetic resonance imaging MRI, which adds some more valuable data to the parameter cloud obtained by the measurement. The scale range for NMR / MRI systems reaches from the macro to the nano scale. Spatial and time resolution do not depend on the scale but rather on the magnetic system, which is also an advantage compared to other measurement methods. 

Recently, NMR and MRI systems themselves have been targets of miniaturization. Meanwhile, benchtop low field NMR systems up to 80MHz resonance frequency (about 1.88T) are available which can easily run in a conventional lab environment, used for stationary or flow-through data achievements. These systems are easy to handle and deliver a bunch of analytical possibilities. Nevertheless, miniaturization went on, and meanwhile developments are reaching into the direction of mini or micro NMR systems [125,127,128,129]. Even wireless systems are now being researched; thus, it can be expected that application of miniaturized and integrated NMR in correlative measurements will be greatly enhanced in the future.

A vision remains of having measurements of different parameters by NMR with the same time resolution, as well as the possibility of measuring all the desired parameters of a process. Thus, combinations of a multitude of analysis instruments will be necessary, ideally within a single system, controlled by a combined measurement electronic system, which internally triggers all methods.
